# Supplementary figures and images for: Eternal-MAML: a meta-learning framework for cross-domain defect recognition
Source: PeerJ Comput Sci. 2025 May 7;11:e2757. doi: 10.7717/peerj-cs.2757 (PMC12190434; doi:10.7717/peerj-cs.2757)

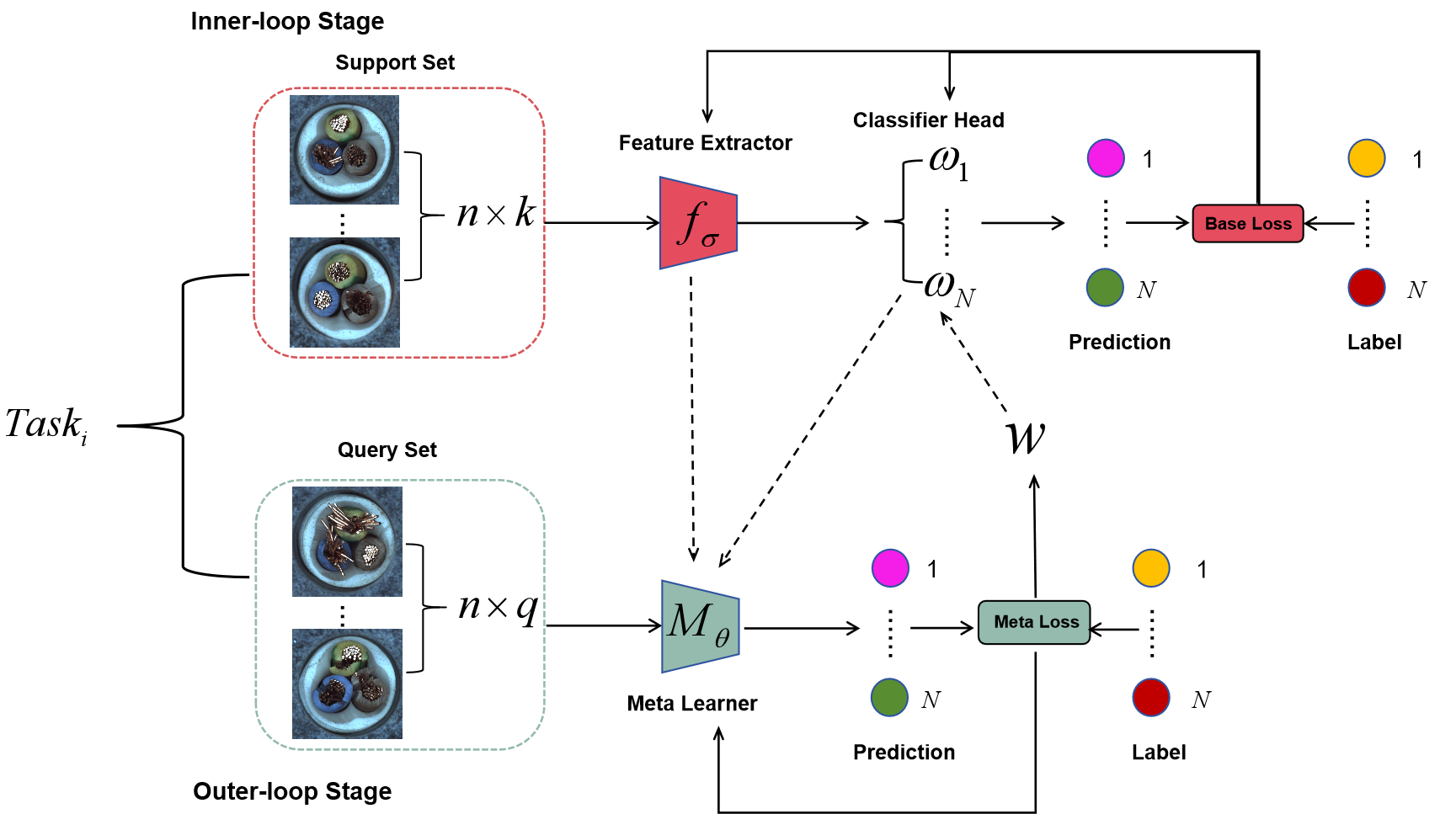

Supplement: Supplemental Information 1 [file peerj-cs-11-2757-s001.zip › Eternal-MAML-main/Framework/eternal-maml.png]
